# The turbulent brain: Modeling vortex interactions for understanding human cognition

**DOI:** 10.1162/NETN.a.554

**Published:** 2026-07-20

**Authors:** Gustavo Deco, Yonatan Sanz Perl, Jianfeng Feng, Morten L. Kringelbach

**Affiliations:** Center for Brain and Cognition, Computational Neuroscience Group, Faculty of Medicine and Life Science, Universitat Pompeu Fabra, Barcelona, Spain; Institució Catalana de la Recerca i Estudis Avançats (ICREA), Barcelona, Spain; International Centre for Flourishing, Universities of Oxford (UK), Aarhus (Denmark), and Pompeu Fabra (Spain); Department of Engineering, Universidad de San Andrés, Buenos Aires, Argentina; Institute of Science and Technology for Brain-Inspired Intelligence, Fudan University, Shanghai, China; Centre for Eudaimonia and Human Flourishing, Linacre College, University of Oxford, Oxford, UK; Department of Psychiatry, University of Oxford, Oxford, UK; Center for Music in the Brain, Department of Clinical Medicine, Aarhus University, Aarhus, Denmark

**Keywords:** Turbulence, Brain dynamics, Oscillators, Hopf, Whole-brain modeling, Cognition

## Abstract

The human brain needs distributed, time-critical computation to efficiently solve complex problems. Turbulence provides such highly efficient spacetime information processing and transmission across widespread brain networks, yet we have been missing a mechanistic understanding of the interactions of turbulent vortices underlying human cognition. Here, we build the first whole-brain model of turbulent vortices as defined by the levels of local synchronization in brain signals quantifying turbulent interactions in vortex space. Specifically, using large-scale human neuroimaging data, we found that the interactions of turbulent vortices is an excellent framework for understanding cognition and brain computation. In particular, we show that when combined with connectome-based predictive modeling, this significantly predict the *g*-factor and the scores on the underlying tasks. In addition, turbulent vortices also distinguish the detailed spacetime dynamics of rest and cognition—and can even distinguish between subtle subcomponents of cognitive tasks, where manipulation of vortices can be shown to change cognition. Overall, this whole-brain framework creates a natural vortex space for the brain computation underlying cognition, as well as potentially providing novel ways of controlling turbulent interactions in disease.

## Author Summary

Turbulence allows for distributed, time-critical computation to efficiently solve complex problems using efficient spacetime information processing and transmission across widespread brain networks. This is the first whole-brain model of turbulent vortices defined by the levels of local synchronization in brain signals quantifying turbulent interactions in vortex space. In large-scale human neuroimaging data, used connectome-based predictive modeling to significantly predict behavior on tasks. In addition, turbulent vortices also distinguish the detailed spacetime dynamics of rest and cognition—and can even distinguish between subtle subcomponents of cognitive tasks, where manipulation of vortices can be shown to change cognition. Overall, this whole-brain framework creates a natural vortex space for the brain computation underlying cognition, as well as potentially providing novel ways of controlling turbulent interactions in disease.

## INTRODUCTION

In people with severe epilepsy, traumatic brain injury and post-traumatic stress disorder (PTSD), seemingly innocuous stimuli like certain smells can lead to a full-blown attack with potential catastrophic impact on the whole-brain dynamics (Hinojosa et al., 2024; Jirsa et al., 2023; Martínez-Molina et al., 2024). Equally, negative experiences can lead to rumination, which can spiral out of control and become clinical depression (Figueroa et al., 2019). These forms of catastrophic impact are consistent with the role of turbulence as a potent form of spatiotemporal chaos that underlies phenomena like of how small perturbations, such as the flapping of butterfly wings, can potentially cause huge systemwide disturbance (Lorenz, 1993). In fact, recent research has demonstrated that the human brain is turbulent (Deco & Kringelbach, 2020; Deco, Leibana Garcia et al., 2023; Deco et al., 2025). Furthermore, it has been demonstrated that measures of whole-brain turbulent dynamics are excellent for predicting the responsiveness to pharmacological treatment in major depressive disorder (Escrichs et al., 2024). Yet, we are still lacking an understanding of why this happens and how to ultimate control this. As such, a whole-brain modeling framework of turbulence found in brain dynamics could be a promising avenue for understanding the underlying dynamics and a first step on the road to controlling turbulence in disease.

Importantly, brain turbulence is not found in the fluids of the brain but rather in the local level of synchronization of brain activity across space and time (Deco & Kringelbach, 2020; Deco, Leibana Garcia et al., 2023; Kawamura et al., 2007; Kuramoto, 1984). This synchronization generates turbulent vortices resembling the whirls from turbulent fluid dynamics (Frisch, 1995; Kolmogorov, 1941a, 1941b) (Bewley et al., 2006; Christoph et al., 2018). Intuitively, any system that has high variability of this local level of synchronization is turbulent and with this comes all the important properties needed for information transfer (Escrichs et al., 2022). Hence, a better understanding of the turbulent dynamics in the human brain could potentially lead to better ways of controlling the system from spinning out of control and become the basis of more effective treatments. Still, reaching this goal requires a deeper understanding of the interactions underlying turbulent dynamics in different brain states.

Here, we build the first whole-brain model of turbulent vortices, defined using different graining partitions of the standard Schaefer brain parcellation (Schaefer et al., 2018) to provide a mechanistic understanding of how their interactions underly the brain computation of human cognition. This can provide a precise description, which eventually could be controlled in much more efficient ways and become the basis of novel treatments.

Still, before such careful control can be implemented, a deeper understanding is needed of the interactions between turbulent vortices underlying normal states of cognition. As we show here, the whole-brain model generates the underlying generative effective connectivity (GEC) in vortex space that when combined with connectome-based predictive modeling (CPM), significantly predicts the participants’ behavioral scores. It also provides a precise way to distinguish different cognitive tasks from rest, as well as distinguishing the more subtle subcomponents of cognitive tasks. We demonstrate that, in principle, manipulation at the level of turbulent vortices can change cognition, that is, from resting to task.

Taken together, the proposed framework of modeling turbulent vortex dynamics is a powerful new approach but also relates to existing methodologies. Neural field models defined over cortical geometry, such as those proposed by Pang and colleagues (Pang et al., 2023), have demonstrated that incorporating the intrinsic shape of the cortical sheet can generate coherent wave-like patterns that account for large-scale fMRI functional connectivity (FC). Notably, these models showed that geometric eigenmodes often outperform connectome eigenmodes in explaining fMRI variability, with the discrepancy diminishing when the structural network is synthesized using a stochastic exponential distance rule. In fact, using the rare long-range anatomical connections in addition to the exponential distance rule provides an even better description of resting-state activity (Vohryzek et al., 2025). Relatedly, recent work on in-strength and out-strength gradients (Koller et al., 2024) has shown that structural embedding produces reliable spatial gradients, such as an in-strength increase from frontal and temporal regions toward parietal areas that can shape the directionality of traveling waves. Other promising approaches computes the curl of the flow of information in human neuroimaging data (Xu et al., 2023).

While these approaches highlight the importance of geometry, connectivity, and gradient structure, they do not characterize the emergent, interacting vortex-like patterns that arise from fluctuations in local synchronization. In our framework, we go beyond the global Kuramoto order parameter, commonly used in EEG, and quantify local synchronization by the local Kuramoto order parameter, which is at the core of turbulence. This allows us to create a direct model of the interactions between turbulent vortices. Please note that previous research based on the turbulent framework has never modeled the vortex space but only turbulence found the traditional fMRI signal space (Deco & Kringelbach, 2020; Deco et al., 2025; Escrichs et al., 2024). Overall, the findings demonstrate that the turbulent vortex space is a natural basis for the brain computation underlying cognition.

## RESULTS

In order to model human cognition, many researchers have examined the fine-grained BOLD signals obtained with fMRI as they solve various tasks. However, using these BOLD timeseries is rarely sufficient for successfully decoding and distinguishing significant aspects of different tasks (Xu et al., 2023). Instead, based on the emergent literature on turbulence in brain dynamics, we came to the idea that a description of the interactions between turbulent vortices could provide a more direct way to model, understand, and control brain dynamics. This intuition is based on the existing research on turbulence in fluid dynamics, where the vortex level provides a convenient basis for modeling, understanding, and control.

**Figure 1.**
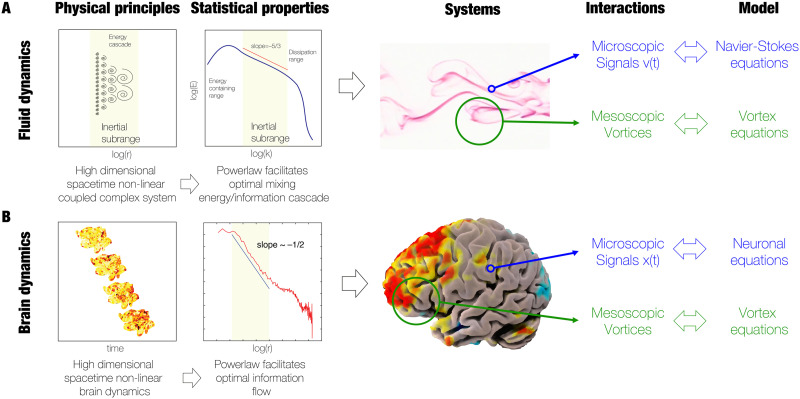
Overview of turbulence in fluids and brain oscillations. (A) Following the discovery and naming of “turbolenza” by Leonardo da Vinci for the whirls of fluids, the science of turbulence was worked out over the next centuries. A significant advance was provided by Kolmogorov’s phenomenological theory of turbulence, which is based on the concept of structure functions, inspired by Richardson’s concept of cascading eddies. This describes the statistical properties of the high dimensional space nonlinear fluid dynamics (left panel). Kolmogorov discovered power laws in an inertial subrange where the structure functions show a universal energy scaling of k−53, where k is the associated wave number of the spectral scale (middle panel). This power law behavior reflects the energy/information transfer cascade found in turbulence. The right panel shows how turbulence can be modelled at the microscopic level by the Navier–Stokes equation but equally at the mesoscopic level by vortex equations, allowing a more efficient description and control of the dynamics. (B) Turbulence is also found in nonfluid systems as demonstrated by Kuramoto, who used coupled oscillators to describe the turbulent whirls of synchronized oscillators promoting optimal mixing. Turbulence is also found in brain dynamics (shown in the flattened brain renderings in left panel), where the phases of brain signal timeseries can be described over time and space by a local Kuramoto order parameter, reflecting the amplitude turbulence. This captures the evolution of the rich variability of the whirls of local synchronization in brain dynamics. The middle panel shows how these turbulent dynamics also follow a power law, reflecting the optimality of efficiency of spacetime information flow in brain dynamics. Here we hypothesize that similar to the principles of fluid dynamics a mesoscopic description of the vortex space will provide a direct and more efficient way to describe and control brain dynamics (right panel).

Figure 1 shows the overall schema of how the principles of turbulence have been firmly established in fluid dynamics at both microscopic and mesoscopic levels (Figure 1A), where power laws have been found that facilitate optimal mixing of the energy/information cascade. Importantly, while the microscopic signals can be described using the Navier–Stokes equations, these are not always computationally tractable. Instead, researchers use the interactions between macroscopic fluid vortices, which also allows them to understand the spacetime dynamics in a more transparent way and to control the levels of turbulence (Protas, 2008; Saffman, 1992).

Similarly, Figure 1B shows how turbulence can also be described using a system of coupled oscillators (Kuramoto, 1984). In particular, this form of turbulence has been found in brain dynamics (Deco & Kringelbach, 2020; Deco, Leibana Garcia et al., 2023; Deco et al., 2025), where there are similar power laws (suggestive of fast information cascades) in the nonequilibrium brain dynamics (Sanz Perl et al., 2023). As shown in the figure, the microscopic signals can be described by neuronal equations such as those established by Hodgkin–Huxley (Hodgkin & Huxley, 1952) but similar to the Navier–Stokes equations, these are also not always computationally tractable. Instead, here, we propose to focus on the mesoscopic vortex space and use a whole-brain model to extract the interactions between turbulent vortices. Like the mesoscopic level in fluid dynamics, the mesoscopic level in oscillatory dynamics could offer a powerful basis for describing the distributed computation underlying cognition.

**Figure 2.**
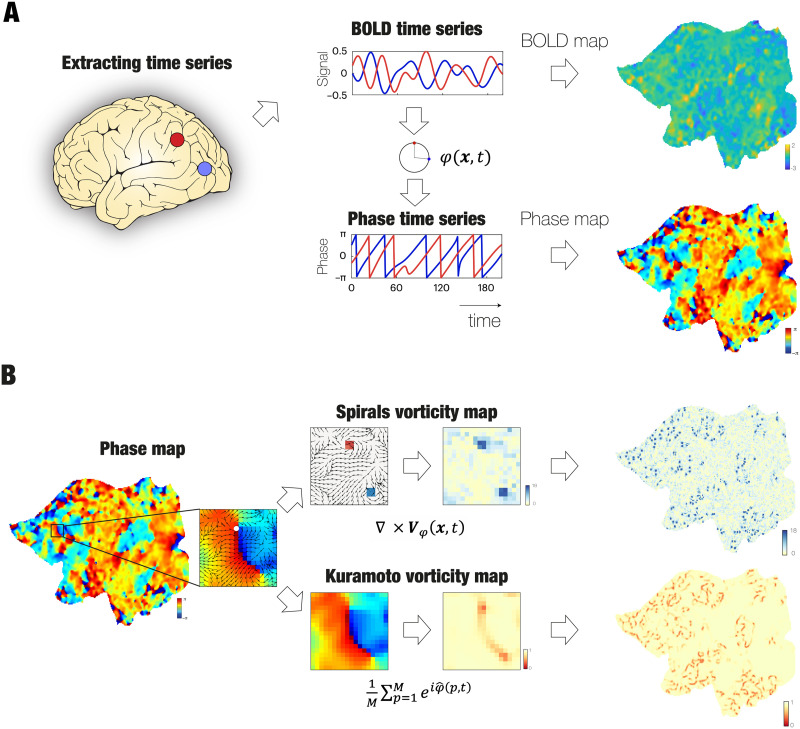
Pipeline for extracting vortices. (A) Neuroimaging provides BOLD time series for each voxel in 3D space. Each voxel in 3D space is transformed to a flattened 2D cortical vertex space and the BOLD time series are transformed into phase time series by using the Hilbert transform. On the left is shown the extraction of the BOLD time series of two brain signals (blue and red, top panel) that are transformed into phase time series (bottom panel). At a given time point, this can be rendered on the flattened surface, where on the top right is shown snapshots of the BOLD values and bottom right the corresponding phase values. (B) There are two ways of extracting vortices from the phase maps: The top middle panel shows the spiral vorticity map resulting from the curl of the flow of the phases. The bottom middle panel shows the Kuramoto vorticity map obtained by directly computing the local Kuramoto order parameters on the phases (see Methods section). The surface renderings show a snapshot of this.

One potential avenue for describing the vortex space was provided by Xu and colleagues who extracted the phases and measured the curl of the flow of information in human neuroimaging data (Xu et al., 2023) (see Figure 2). They found that the resulting spiral-like, rotational wave patterns are widespread during both resting and cognitive task states, propagating across the cortex while rotating around their phase singularity centers, giving rise to spatiotemporal activity dynamics with nonstationary features. They demonstrated that these properties of brain spirals provide sensitive measures that can be used to classify different cognitive tasks.

However, it is not easy to derive a model of brain spiral dynamics for quantifying their interactions. At the same time, these brain spirals are intimately linked to turbulent vortices (Deco & Kringelbach, 2020; Deco et al., 2023; Deco et al., 2025) (see Figure 2B). Indeed, it is straightforward to build a model of coupled oscillators underlying turbulence, given that this research already uses the local Kuramoto order parameter (Kawamura et al., 2007) to measure the level of turbulence in oscillatory systems such as the brain (Deco & Kringelbach, 2020; Deco, Leibana Garcia et al., 2023; Deco et al., 2025). In fact, the use of Kuramoto oscillators creates a natural interpretation for measuring the flow between turbulent vortices and, importantly, allowing for direct modeling of the interactions between vortices.

In a technical tour de force, here, we created a vortex space from the fine-grained vertex space created by neuroimaging BOLD signals, where a vertex is the projection of a 3D brain voxel to the cortical surface. From this space, the interactions between each vertex can be reduced to a more coarse-grained parcellation of 1,000 regions (Schaefer1000) to an even more coarse-grained partition of 100 regions (Schaefer100). This is achieved using an influential partition technique (Snyder et al., 2020), effectively creating a lower dimensional vortex space, where an explicit equation can be derived for the modeling of the interactions between the 100 vortices.

This vortex space can be used as the basis of a whole-brain model that can quantify the interactions between vortices in vortex space. In other words, this framework is focusing on the vortex space rather than the signal vertex space obtained from BOLD measurements. This framework is thus moving away from modeling signal vertex space to modeling vortex space, which is crucial to capture the essential elements of the information cascade in turbulence. Excitingly (as shown in the Methods section), modeling the interactions in vertex space with coupled Kuramoto local oscillators naturally leads to the modeling the vortex space with the celebrated Stuart–Landau equation (sometimes also called the Hopf equation), which is universally used in physics to measure most if not all physical phenomena. As such the research follows the dictum of Feynman who wrote “What I cannot create I do not understand,” which emphasizes the importance of modeling for understanding any physical phenomenon.

### Comparing Kuramoto Versus Spiral Vorticity Over Time and Space

**Figure 3.**
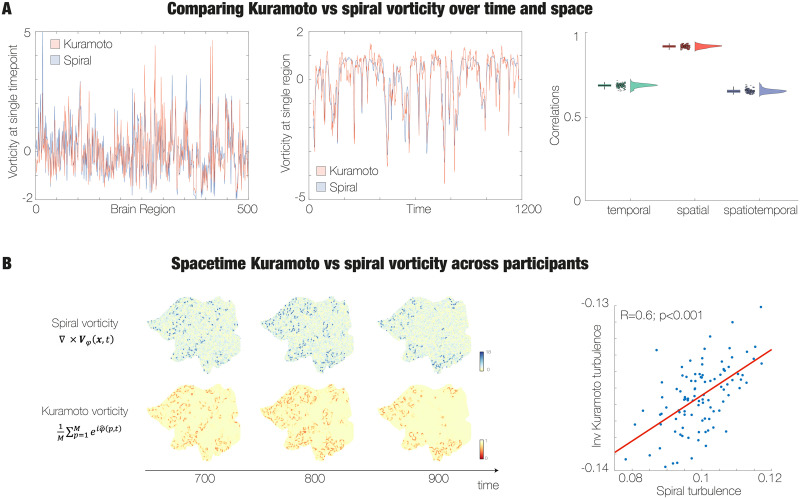
Equivalence between measures of Kuramoto and spiral vorticity. (A) The left graph is comparing the measures of Kuramoto (red) and spiral (blue) vorticity. In order to show the similarity of the two measures, we z-scored the average of the values at a single time point in the vertices within each of the parcels of the Schaefer1000 parcellation. Please note that Kuramoto values are inverted to improve the visualization of the similarities. Here, the graph only shows these values for one hemisphere. Similarly, the middle panel is showing the z-scored values of a single parcel over the full time (1,200 TRs). The right panel is showing a boxplot of the correlations between Kuramoto and spiral vorticity across time (green), space (red), and spacetime (blue) for a subset of 100 HCP participants. Please note the generally high correlations across all three, with the highest correlation found in the spatial domain. (B) The left panel visualizes the equivalence between spiral and Kuramoto vorticity by showing three snapshots of the vorticity in the full vertex space of an individual. This high spatiotemporal correlation can also be observed across participants, using the measures of spiral and Kuramoto turbulence. The graph shows the high correlation between both measures of turbulence.

As stated above, there is intuitively a close link between brain spirals and Kuramoto vorticity. In order to quantify this intuition, we directly compared the two (Figure 3). Following Xu and colleagues (Xu et al., 2023)—and as shown in the Methods section—we used the definition of brain spirals in vertex space as the curl of the flow of the phases (Equation 2). We compared this with the local order Kuramoto parameter (Equation 4) computed in the Schaefer1000 parcellation and compared with brain spirals in this space (obtained by averaging the corresponding values of the vertices within each parcel).

Figure 3A (left panel) shows the results of comparing the measures of Kuramoto (red) and spiral (blue) vorticity across one hemisphere of 500 parcels. Note that the two measures are inverted in terms of measuring synchronization, that is the lowest level of synchronization is found at the center of the brain spiral, while the opposite is true for Kuramoto vorticity (compare Equations 2 and 4). Therefore, in the graph we have inverted the Kuramoto vorticity values and z-scored both values to improve the visualization of their similarities. In the middle graph, the figure shows the similarity in terms of time by showing the z-scored values of a single parcel over the full time (1,200 TRs (repetition time)). The right panel shows boxplots as quantification of the correlations between the two measures in terms of time (green), space (red), and spacetime (blue) for a subset of 100 Human Connectome Project (HCP) participants. The highest correlation is found in the spatial domain.

Figure 3B shows these spatial similarities very clearly through three snapshots of the two kinds of vorticity (spiral vorticity in green and Kuramoto vorticity in yellow) in the full vertex space in an individual. In order to further quantify these similarities, we also computed the spiral turbulence (Equation 3) and the Kuramoto turbulence (Equation 5). As can be seen from the scatterplot in the right panel, this high spatiotemporal correlation is also found across the 100 participants (*R* = 0.57, *p* < 0.001).

Although spiral vorticity and the local Kuramoto order parameter are empirically correlated, they are not mathematically equivalent and capture distinct aspects of local phase dynamics. Spiral vorticity is derived from spatial phase gradients and quantifies the local curl of phase flow, making it sensitive to the geometric organization and directionality of rotational activity. In contrast, the local Kuramoto order parameter measures the degree of phase coherence within a neighborhood, independent of spatial orientation or phase gradients. Consequently, the two measures can diverge: Rotational phase flows with local phase dispersion may exhibit high spiral vorticity but low synchrony, whereas highly synchronized yet spatially homogeneous phase patterns can yield high Kuramoto order with negligible spiral vorticity. Their observed correlation therefore reflects complementary sensitivity to local collective organization rather than circularity. Crucially, this partial correspondence enables a coarse-graining approach in which vortex dynamics can be described in terms of Kuramoto-based variables, allowing us to directly quantify interactions between vortices and derive effective dynamical equations in vortex space.

### Whole-Brain Modeling Using Partition

Having shown the equivalence between spiral and Kuramoto vorticity, we proceed to model interactions between the Kuramoto vortices. In order to do so, we simplified the problem through using a common method for space partitioning (Snyder et al., 2020), creating a lower dimensional vortex space that can be used for modeling. We demonstrated the effectiveness of this approach from first principles by deriving the whole-brain model in Schaefer100 vortex space from a phase model in the finer Schaefer1000 partition.

Please note that the choice of parcellation is not arbitrary in the present framework but constrained by the analytical coarse-graining procedure used to derive the vortex-space dynamics. Specifically, our derivation requires a coarse partition that is a strict subpartition of the finer-scale network in order to express cluster order parameters and obtain the reduced vortex-space equations. As a result, only parcellation schemes that are hierarchically defined can be used consistently within this framework. The Schaefer atlas is explicitly constructed to satisfy this requirement, making the Schaefer-1000 to Schaefer-100 mapping a natural and analytically consistent choice (see details and parameters selection in the Methods section).

**Figure 4.**
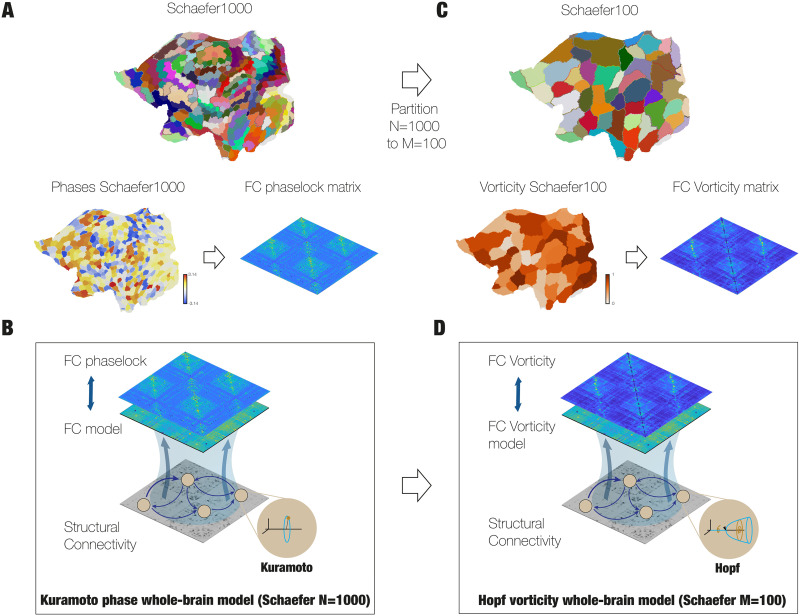
Whole-brain model in vortex space derived through space partitioning. A whole-brain model in vortex space can be derived from first principles from a phase model in a finer partition. (A) First, for the whole-brain model in phase space, we use the fine Schaefer1000 partition (upper left part) to extract the corresponding phase lock matrix from the averaged signals from the empirical data in vertex space. (B) A whole-brain model was built using a Kuramoto model for the local dynamics and coupled through the structural connectivity matrix for fitting the empirical phase lock matrix. (C) Importantly, this model with Kuramoto local dynamics for the phases directly leads to an analytical whole-brain model with Stuart–Landau local dynamics of the vortex space (see Methods section). Specifically, we define this Schaefer100 parcellation (top) as a partition of the Schaefer1000, where each parcel contains the Kuramoto vorticity (bottom left). The FC of this Kuramoto vorticity can then be used to fit the vortex whole-brain model. (D) As shown the vortex whole-brain model is using the analytical version of the Hopf equation, now derived from first principles.

This partitioning is schematized in Figure 4. First, we show renderings of the Schaefer1000 parcellation on a flattened hemisphere (top) as well as the phases of BOLD signals giving rise to the FC phaselock matrix (Figure 4A). This is used in a whole-brain model of the FC (functional connectivity) phaselock matrix using the Kuramoto phase model for the local dynamics (Cabral et al., 2014) (Figure 4B). Using the partitioning method we can derive a closed equation for the vortices in the coarser Schaefer100 vortex space. Figure 4C shows renderings of the Schaefer 100 parcellation in a flattened hemisphere (top) and with the values of Kuramoto vorticity in this parcellation (bottom left), giving rise the FC vorticity matrix (bottom right). As shown in Figure 4D, this is then used in a whole-brain model derived analytically from finer parcellation Kuramoto model to produce a Hopf model in this coarser parcellation of the vortex space.

### Comparing Hopf Vortex Model Versus Kuramoto Phase Model

**Figure 5.**
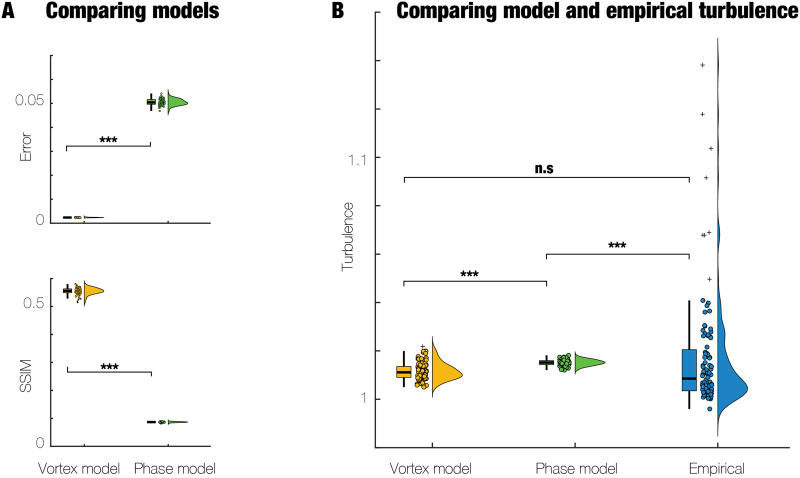
Hopf vortex whole-brain model is better than Kuramoto phase model for fitting empirical data. We compared the fit of both models in terms of the error (top) and SSIM (bottom) between the generated and empirical FC in vortex space. The Hopf vortex model is already in vortex space but the Kuramoto model is in signal space. So, in order to directly compare the accuracy of the two models, the Kuramoto model must be in vortex space. This is achieved by a two-step process: (a) optimize the Kuramoto phase model to the empirical phase lock data and (b) use this optimized Kuramoto phase model to generate time series and compute the corresponding FC in vortex space (see Methods section). (A) The figure clearly shows that the Hopf vortex model significantly outperforms the Kuramoto phase model. (B) Similarly, the Hopf vortex model was significantly better than the Kuramoto phase model in fitting the level of empirical turbulence. Importantly, there were no significant differences between the vortex model and the empirical data, which was not the case for the phase model.

Figure 5 shows a direct comparison of the two models in vortex space. We measured this in terms of the fit of both models for the error (top) and structural similarity index measure (SSIM; bottom) between the generated and empirical FC in vortex space. To directly compare the two measures, we transformed the Kuramoto model from signal space to vortex space through a two-step process: (a) optimize the Kuramoto phase model to the empirical phase lock data and (b) use this optimized Kuramoto phase model to generate time series and compute the corresponding FC in vortex space (see Methods section).

Figure 5A shows that the Hopf vortex model significantly outperforms the Kuramoto phase model for the error (Wilcoxon *p* < 0.001) and SSIM (Wilcoxon *p* < 0.001). Similarly, Figure 5B shows that the Hopf vortex model was significantly better than the Kuramoto phase model for fitting the level of empirical turbulence (Wilcoxon *p* < 0.001). In fact, there were no significant differences between the turbulence of the data generated by the vortex model and turbulence of the empirical data. In contrast this was not true for the phase model, demonstrating the superiority of the vortex model over the phase model.

### Cognition Can Be Described by Whole-Brain Model of the Interactions of Vortices

As mentioned in the introduction, many attempts have been made to find adequate measures for successfully decoding and distinguishing significant aspects human cognitive processing. Here, we were able to show that whole-brain models of the interactions between vortices can be very useful for classification and understanding the mechanisms for the underlying computation.

**Figure 6.**
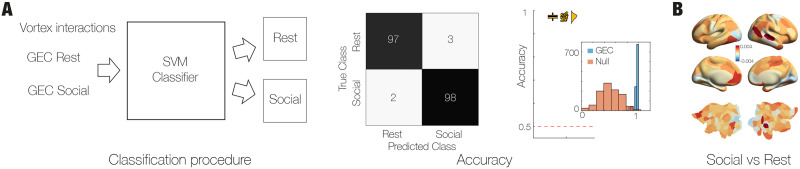
Classification of vortex interactions is highly efficient for distinguishing rest versus social task. Using the Hopf vortex whole-brain model can be used to quantify the vortex interactions in the form of the GEC for different conditions (rest and SOCIAL task). (A) We classified the individual GECs corresponding to rest and social for all 971 participants and found a near perfect classification on the generalization set with 0.975 ± 0.025 (*M* ± *SD*) accuracy on the 1,000-folds. This is shown in the confusion matrix and in the boxplot across the folds. We assessed the significance of the classifier performance by compared with a. null model obtained by training the same classifier but with the label scrambled 1,000 times. The histograms show the performance of both classifiers across the 1,000 repetitions. We obtained a highly significant (*p* = 0.002) by computing an empirical *p* value by counting the number of times that the null models outperform the GEC model. (B) Rendering of the differences in regional degree of the average GEC for rest and SOCIAL task.

Figure 6 shows the results of using the Hopf vortex whole-brain model to quantify the vortex interactions. In particular, the model provides an optimized individual measure of the GEC matrix, which is a direct measure of the interactions between vortices (see Methods section). This is then used on two different conditions of resting state and SOCIAL task. For this we used support vector machine (SVM) classification with the individual GECs for both conditions for all 971 HCP participants. This showed a near perfect classification on the generalization set with 0.975 ± 0.025 (*M* ± *SD*) accuracy on the 1,000-folds. This is also shown in the confusion matrix and in the boxplot across the folds (Figure 6A). The differences in regional degree of the average GEC for rest and SOCIAL task are shown in brain renderings (Figure 6B).

**Figure 7.**
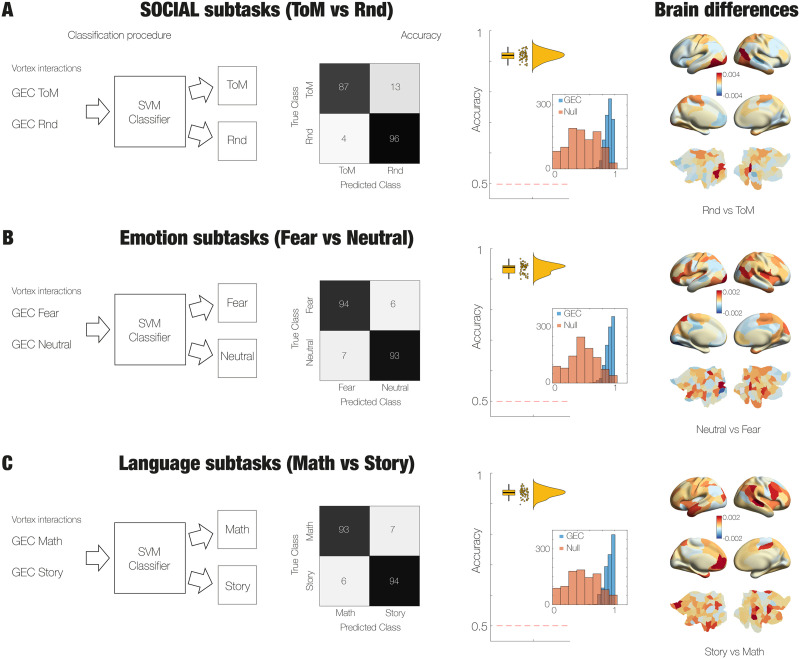
Interactions in turbulent vortex space are highly accurate in classifying subtle subtasks. Similar to Figure 6, we used the Hopf vortex whole-brain model to quantify the vortex interactions in the form of the GEC for different subtle subtask conditions. (A) Within the SOCIAL task, which uses moving dots on a screen, there are two conditions, namely moving dots evoking theory of mind (ToM) and randomly moving dots (Rnd). We found a near perfect classification on the generalization set with 0.92 ± 0.02 (*M* ± *SD*) accuracy and significant with respect to the null model (*p* = 0.01). The histograms show the performance of the classifiers and null models across the 1,000 repetitions, as in Figure 6. The rendering shows the differences in regional degree of the average GEC for ToM versus Rnd subtasks. (B) Same but for the Fear and Neutral subtasks of the EMOTION task. We found a near perfect classification on the generalization set with 0.94 ± 0.02 (*M* ± *SD*) accuracy and significant with respect to the null model (*p* = 0.01). The rendering shows the differences in regional degree of the average GEC for Fear versus Neutral subtasks. (C) Similar but for the Maths and Story subtasks of the LANGUAGE task. We found a near perfect classification on the generalization set with 0.94 ± 0.02 (*M* ± *SD*) accuracy and significant with respect to the null model (*p* = 0.01). The rendering shows the differences in regional degree of the average GEC for Maths versus Story subtasks.

Importantly, this whole-brain modeling is not just useful for distinguishing tasks from rest but is also highly accurate in classifying the subtleties between subtasks. Figure 7 shows three examples of using the Hopf vortex whole-brain model to quantify the vortex interactions in the form of the GEC for different subtle subtask conditions.

Figure 7A shows the near perfect classification on the generalization set with 0.92 ± 0.02 (*M* ± *SD*) accuracy of comparing the two conditions in the SOCIAL task. This task uses moving dots on a screen with two distinct conditions of the moving dots either evoking ToM or moving randomly (Rnd). The brain renderings show the differences in regional degree of the average GEC for ToM versus Rnd subtasks.

Figure 7B shows the results of doing the same comparison for the Fear and Neutral subtasks of the EMOTION task. Again, a near perfect classification was found on the generalization set with 0.94 ± 0.02 (*M* ± *SD*) accuracy. The brain rendering shows the differences in regional degree of the average GEC for Fear versus Neutral subtasks.

Finally, Figure 7C shows the results of classifying the Maths and Story subtasks of the LANGUAGE task. A near perfect classification was found on the generalization set with 0.94 ± 0.02 (*M* ± *SD*) accuracy. The brain rendering shows the differences in regional degree of the average GEC for Maths versus Story subtasks.

These results leave open the intriguing possibility of controlling the turbulent brain dynamics in vortex space, similar to how fluid dynamics can be efficiently controlled in vortex space. In a proof of principle, we were able to find model perturbations in vortex space that led to a transitioning between resting and the SOCIAL task. Similar to the perturbation strategy we have previously used in signal space (Deco et al., 2019), here, we used noise perturbations of the local dynamics of the Hopf model in vortex space to force a transition between resting and SOCIAL task. We were able to find a set of perturbation that transformed the dynamics of the model resting state to the SOCIAL task with a model fit correlation of 0.67 to the empirical FC of the SOCIAL task. While this is promising, more work is clearly needed in the future.

### Predicting Behavior From Modeling of Interactions Between Turbulent Vortices

In order to assess the behavioral relevance of the vortex-space interactions captured by the GEC (GEC Vortex), we applied CPM (see Methods section) (Greene et al., 2018; Shen et al., 2017) to predict the *g*-factor of intelligence (Dubois et al., 2018) and four of the main underlying performance scores across cognitive domains: Card Sorting (characterizing executive functions), Picture Vocabulary (crystallized intelligence), Progressive Matrices (PMAT24 (Penn Progressive Matrices, 24-item version), fluid intelligence), and Processing Speed (speed of cognition).

**Figure 8.**
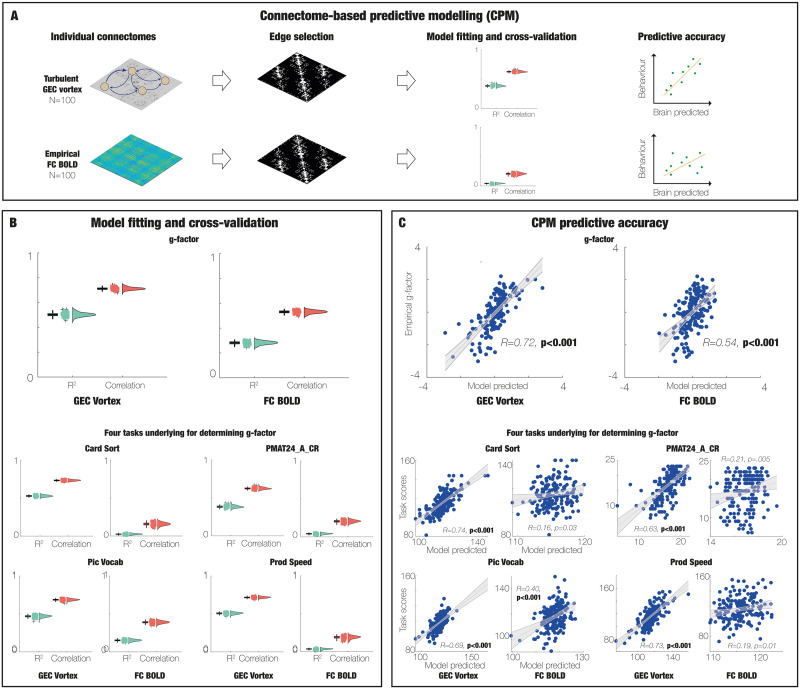
Predicting behavior from modeling of turbulent vortex space interactions. (A) As predictors of the *g*-factor of intelligence and four of the main scores in different cognitive domains (Card Sort, Pic Vocab, PMAT24, and Prod Speed), we applied CPM using the two different matrices, either the GEC Vortex (generated by the model in vortex space) or the FC BOLD matrix (from the empirical BOLD signals). In accordance with standard CPM procedure, we performed a feature selection considering the full data set and then we build ridge models for each case. We evaluated each model by implementing 100 repetitions of a 10-fold outer cross-validation framework. Finally, we compared the predicted brain behavior output with the actual scores. (B) Repeating this procedure 100 time in each case, the subplot shows the boxplots of between the observed and predicted task scores and the *R*^2^ of the model for the *g*-factor (top) and for the four tests (below) with GEC Vortex (left) and FC BOLD (right). Importantly, the CPM with GEC Vortex outperforms the FC BOLD model for all the cases and metrics. (C) Attesting to this, the subplot shows the scatter plots comparing the predicted and the observed behavior for each case. Please notice how the GEC Vortex are significantly better at predicting task scores (highlighted in bold) compared with the FC BOLD.

We constructed two predictive models from the GEC Vortex matrices and the conventional FC BOLD matrices. We implemented the CPM following standard procedures for each of the four cognitive domain and for each of feature space (see Methods section). Briefly, as outlined in Figure 8A, we performed a feature selection considering the full data set and then we build ridge models for each case. We evaluated each model by implementing 100 repetitions of a 10-fold outer cross-validation framework. Finally, we compared the predicted brain behavior output with the actual scores. We evaluated the performance of each model by comparing the *R*^2^ of the model and the correlation between the predicted and the actual behavior score across 100 repetitions of the full procedure (Figure 8B). Importantly, as can be seen in the figure, across all cognitive domains and evaluation metrics, the models based on GEC Vortex consistently significantly outperformed those based on conventional FC BOLD. Specifically, higher correlations between observed and predicted behavioral scores, as well as larger *R*^2^ values, were observed for GEC Vortex-based models for *g*-factor and all four tasks. This can be seen very clearly in the scatter plots in Figure 8C comparing predicted and observed scores, revealing tighter associations and reduced dispersion for GEC Vortex predictions relative to FC BOLD-based predictions.

Taken together, these findings demonstrate that modeling vortex-space interactions using GEC Vortex captures behaviorally meaningful information beyond that contained in standard FC BOLD, highlighting the relevance of turbulent vortex dynamics for understanding individual differences in human cognition.

## DISCUSSION

Here, we have shown that whole-brain modeling of the interactions of turbulent vortices provides an excellent framework for quantifying and understanding brain dynamics. This is important since turbulence is a fundamental principle found at all scales in Nature, from the microscopic scale to the scale of the universe. The recent discovery of turbulence in brain dynamics provides a mechanistic basis of highly efficient time-critical information processing and transmission across wide-spread brain networks. Here, we have provided the first evidence that the interactions between turbulent vortices could be a fundamental principle of brain computation.

We quantified these interactions through building a whole-brain model of the vortex space and found that when combining the GEC with CPM, this significantly predict the *g*-factor and the scores on the tasks that contribute to this unifying measure. In addition, the GEC also successfully distinguishes the detailed spacetime dynamics of rest and cognition. It can even help distinguish between subtle subcomponents of cognitive tasks. Importantly, this Hopf whole-brain model in vortex space was derived from first principles from the fine-grained vertex space of interacting coupled Kuramoto phase oscillators. Overall, this provides a natural vortex space for brain computation and could potentially offer new ways of controlling the breakdown in disease.

The whole-brain modeling in vortex space was made possible by providing a mapping between the signal space in a fine-grained space of 32k vertices to a coarser-grained vortex space of 100 regions. For the necessary partitioning scheme, we used the well-known method by Snyder and colleagues (Snyder et al., 2020), which made it possible to derive analytically the whole-brain model of the vortex space from using a model with the Kuramoto phase oscillator in the vertex space. We were able to show that this results in a model using the Hopf (or Stuart–Landau) oscillator for the local dynamics in vortex space.

This result can be seen in the wider context of the use of Hopf whole-brain models, which, set at the edge of the bifurcation point and coupled to anatomical connectivity of the connectome, have been shown to be able to model the signal space of brain dynamics (Deco et al., 2017). The Hopf model is highly efficient for capturing the stochastically fluctuating signal with oscillatory components found in the slow dynamics of BOLD signals. The reason for this ability can be found in the fact that a whole-brain model using local dynamics of the exact mean-field model with inhibitory and excitatory integrate-and-fire neurons lead to similar dynamics as the Hopf model, poised just below the critical Hopf bifurcation (Perl et al., 2023). Furthermore, Piccinini and colleagues found that from all possible connectome-based models, human fMRI was best described by a Hopf model just below the bifurcation point (Piccinini et al., 2022). This matches the conclusions of Sip and colleagues (Sip et al., 2023) who found that the regional and network-level dynamics of resting-state fMRI can be described as noisy fluctuation around a single fixed point, which again is best captured by the Hopf model. As such, this body of research has convincingly shown the Hopf model is superior to what is often termed as more biologically detailed models of excitation and inhibition.

Still, the physics of fluid turbulence has shown that the best models are not based at the signal level but rather at the higher vortex level. The rotationality of the vortices provide sufficient information to understand and ultimately control turbulence. Our results provide a similar reason to move beyond the signal level for the oscillatory turbulence found in brain dynamics. The partitioning method used here clearly demonstrates that the Hopf whole-brain model emerges as the natural framework for modeling brain turbulence. In particular, using the vortex space for the whole-brain model provides an individualized an explicit description of the interactions of the brain vorticity through the GEC.

The results here show the utility of this individualized GEC since it is excellent not only for classification of rest versus SOCIAL task but also for distinguishing between very similar subphases within tasks. However, this is not just useful for classification but also provides the necessary elements for a mechanistic region-level explanation of task computation. Take for example the differences between the interactions during resting state and performing the SOCIAL task of assigning meaning to dots moving around a computer screen and deciding whether these reflect some degree of ToM or simply random movement. Here, there are distinct differences in the brain networks engaged, with clear differences in the task related activity in visual regions and in the medial prefrontal cortex for rest and SOCIAL task.

Over the past decade, a substantial literature has characterized how brain activity reorganizes when individuals transition from spontaneous rest to active task engagement, providing a crucial context for interpreting our framework. A series of influential papers by Cole, Ito, and collaborators have shown that FC does not remain static across cognitive contexts but instead undergoes systematic and task-specific reconfiguration. Their work demonstrates that large-scale control networks flexibly adjust their coupling patterns to support distinct computational demands, thereby revealing a dynamic functional architecture that differentiates rest from a wide variety of tasks (Cole et al., 2014; Ito et al., 2017). Building on this functional perspective, Yan et al. (2021) demonstrated that these rest–task differences are not arbitrary; rather, they preferentially follow distinct subnetworks carved out by the brain’s structural connectivity. This mapping suggests that the anatomical connectome provides a scaffold that channels task-related reorganization along specific structural pathways.

More recently, studies using ordinary differential equation (ODE) modeling have leveraged dynamical-systems theory to classify rest and task states based on their underlying temporal evolution. These models have revealed that cognitive engagement alters characteristic dynamical motifs—such as attractor landscapes, stability profiles, and flow architectures—that allow for a precise discrimination between the two states (Singh et al., 2020; Kashyap et al., 2025). Together, this body of work has established that rest–task differences can be detected through changes in FC, through their embedding in the structural connectome, and through shifts in dynamical regimes. Even more, recent studies have shown that these changes in dynamical regimes are associated with different levels of nonequilibrium. Task-evoked activity operates farther from thermodynamic equilibrium compared with resting state (Deco et al., 2022; Deco, Sanz Perl, et al., 2023; Lynn et al., 2021).

Our model of vortex interactions contributes a novel and complementary layer to this ongoing discourse. Instead of focusing exclusively on connectivity or structural constraints, we describe how rest and task states differ in terms of the spatiotemporal organization of turbulent vortices, the fundamental dynamical events that shape information flow in our framework. We show that the configuration, interactions, and propagation of these vortices provide a natural basis for distinguishing resting from task-evoked activity, as well as discriminating more subtle subcomponents within task states. In this way, our framework unifies and extends earlier findings by grounding rest–task differences in the generative mechanisms that produce them, offering a mechanistic account of how cognition emerges from, and modulates, the underlying turbulent dynamics of the human brain.

Overall, modeling the interactions between turbulent vortices is a powerful way of understanding brain dynamics in health. But equally, this also opens the possibility of controlling brain dynamics in disease. Excitingly, in a proof of principle, we were able to perturb the whole-brain model of resting state to instead fit the SOCIAL task condition with a high level of precision. This opens up for a much needed ability to control turbulence in disease. Future studies could investigate data from patients with, for example, traumatic brain injury, PTSD, and depression to design therapeutic interventions to stop full-blown attack with potential catastrophic impact on the whole-brain dynamics (Figueroa et al., 2019; Hinojosa et al., 2024; Jirsa et al., 2023; Martínez-Molina et al., 2024) by changing the interactions between vortices to a healthy regime. Longer-term, this could have important impact on providing new therapies for current treatment-resistant neuropsychiatric disorders.

## METHODS

### Empirical fMRI Data

#### Neuroimaging ethics HCP.

The Washington University–University of Minnesota Consortium obtained full informed consent from all participants, and research procedures and ethical guidelines were followed in accordance with Washington University institutional review board approval (Mapping the Human Connectome: Structure, Function, and Heritability; IRB # 201204036).

#### Neuroimaging HCP participants.

The data set used for this investigation was selected from the March 2017 public data release from the HCP where we chose a sample of 971 participants with data from resting state and all seven cognitive tasks.

#### Neuroimaging acquisition for fMRI HCP.

The 971 HCP participants were scanned on a 3-T connectome-Skyra scanner (Siemens). We used one resting state fMRI acquisition of approximately 15 min acquired on the same day, with eyes open with relaxed fixation on a projected bright crosshair on a dark background as well as data from the seven tasks. The HCP website (https://www.humanconnectome.org/) provides the full details of participants, the acquisition protocol and preprocessing of the data for both resting state and the seven tasks.

#### Preprocessing and extraction of functional timeseries in fMRI resting data.

The preprocessing of the HCP resting state and task datasets is described in details on the HCP website. Briefly, the data are preprocessed using the HCP pipeline, which is using standardized methods using FSL (FMRIB (Oxford Centre for Functional MRI of the Brain) Software Library), FreeSurfer, and the Connectome Workbench software (Glasser et al., 2013; Smith et al., 2013). This preprocessing included correction for spatial and gradient distortions and head motion, intensity normalization and bias field removal, registration to the T1-weighted structural image, transformation to the 2 mm Montreal Neurological Institute (MNI) space, and using the FIX (FMRIB's ICA-based X-noiseifier) artefact removal procedure (Navarro Schröder et al., 2015; Smith et al., 2013). The head motion parameters were regressed out and structured artefacts were removed by ICA (Independent Component Analysis) + FIX processing (Independent Component Analysis followed by FMRIB’s ICA-based X-noiseifier) (Griffanti et al., 2014; Salimi-Khorshidi et al., 2014). Preprocessed timeseries of all grayordinates are in HCP CIFTI (Connectivity Informatics Technology Initiative) grayordinates standard space and available in the surface-based CIFTI file for each participants for resting state and each of the seven tasks.

We used a custom-made MATLAB script using the ft_read_cifti function (Fieldtrip toolbox) (Oostenveld et al., 2011) to extract the average timeseries of all the grayordinates in each region of the Schaefer parcellation, which are defined in the HCP CIFTI grayordinates standard space. Furthermore, the BOLD time series were transformed to phase space by filtering the signals in the range between 0.008–0.08 Hz, where we chose the typical high-pass cutoff to filter low-frequency signal drifts (Fox et al., 2005), and the low-pass cutoff to filter the physiological noise, which tends to dominate the higher frequencies. We then applied the Hilbert transforms in order to obtain the phases of the signal for each brain node as a function of the time.

### The HCP Task Battery of Seven Tasks

The HCP task battery consists of seven tasks: working memory, motor, gambling, language, social, emotional, and relational, which are described in details on the HCP website (Barch et al., 2013). HCP participants performed all tasks in two separate sessions (first session: working memory, gambling and motor; second session: language, social cognition, relational processing, and emotion processing).

### Schaefer Parcellations

Schaefer and colleagues created a publicly available population atlas of cerebral cortical parcellation based on estimation from a large data set (*N* = 1,489) (Schaefer et al., 2018). They provide parcellations of 100 and 1,000 areas available in surface spaces, as well as MNI152 volumetric space. We used here the Schaefer parcellations with 1,000 and 100 parcels and extracted the timeseries from HCP using the HCP surface space version.

### Structural Connectivity Using dMRI

The HCP database contains diffusion spectrum and T2-weighted imaging data from 32 participants with the acquisition parameters described in detail on the HCP website (Setsompop et al., 2013). The freely available Lead-DBS software package (https://www.lead-dbs.org/) provides the preprocessing, which is described in detail in Horn and colleagues (Horn et al., 2017), but briefly, the data were processed using a generalized q-sampling imaging algorithm implemented in DSI studio (https://dsi-studio.labsolver.org). Segmentation of the T2-weighted anatomical images produced a white-matter mask and co-registering the images to the b0 image of the diffusion data using SPM12. In each HCP participant, 200,000 fibers were sampled within the white-matter mask. Fibers were transformed into MNI space using Lead-DBS (Horn & Blankenburg, 2016). We used the standardized methods in Lead-DBS to produce the structural connectomes for both Schaefer1000 and Schaefer100 parcellation schemes.

### Theoretical Turbulent Vortices Framework

#### Spiral vorticity.

Generating the spiral vortices requires computing the phase map of the empirical fMRI signals in the 2D flattened cortical space and then calculating the phase vector field, that is, the phase velocity flow, whose curl defines the spirals vorticity. These spirals characterize local rotational motion near a specific point, that is, the tendency to rotate, as observed from above at that point and moving with the flow. For a subset of 100 participants of the HCP database described above, we consider 2-mm standard CIFTI grayordinate space of half hemisphere comprising 32k cortical vertices (vertex space), which are represented in the 2D flattened cortex surface. Temporal filtering of the BOLD fMRI signals is done by applying the second-order zero-phase Butterworth filter (0.008 Hz < f < 0.08 Hz) to each voxel of the cortex to focus on slow neuronal fluctuations. After this, the data are smoothed by performing a Gaussian spatial filtering with a width of 4 mm. Finally, for each time point *t*, we compute the phase map *φ*(***x***, *t*) in the 2D vertex space ***x*** using the Hilbert transform. The phase vector field is computed as the spatial gradient of the phase map given by:

$$V_{\varphi }\left(x,t\right)=\nabla \varphi \left(x,t\right)$$ (1)

The spatial gradient was determined by taking derivatives across the two spatial dimensions, numerically computed by using central finite differences. Circular statistics were used to compute the differences. Finally, we derived the spiral vorticity in vertex space by computing the spatial curl of the phase vector field, that is:

$$\Omega \left(x,t\right)=\nabla \times V_{\varphi }\left(x,t\right)$$ (2)

The curl is thresholded at Ω > 1 or Ω < −1 to find the potential core of spirals with very high vorticity. We define spiral based turbulence *T*_*spiral*_ as the standard deviation of the spiral vorticity map across space and time:

$$T_{\text{spiral}}=\langle \Omega {\left(x,t\right)}^{2}\rangle -{\langle \Omega \left(x,t\right)\rangle }^{2}$$ (3)

where the brackets 〈 〉 denotes average across vertex space and time.

Importantly, the curl must be computed on the phase-based flow field, not on the raw BOLD signal, because only the phase encodes the direction and propagation of neural activity (Xu et al., 2023). Turbulence, vorticity, and rotational wave dynamics are defined in physics exclusively from the phase of an oscillatory field, not its amplitude. This is the reason why Kuramoto defined turbulence in the phase space and not in amplitude space (Kuramoto, 1984). In BOLD fMRI, intensity fluctuations do not constitute a physical flow, apparent “motion” of amplitude is dominated by hemodynamics, smoothing, and noise, and does not reflect directional propagation. In contrast, the phase field provides a well-defined velocity vector describing how cortical waves travel across the brain, and the curl of this velocity yields a meaningful measure of rotational dynamics analogous to vortices in fluids, spiral waves in excitable tissue, and traveling-wave models of cortical activity.

#### Kuramoto vorticity.

As has been shown, turbulence is not restricted to fluid dynamics but is also found in other physical systems including coupled oscillators (Kawamura et al., 2007) and brains (Deco & Kringelbach, 2020; Deco, Leibana Garcia et al., 2023; Deco et al., 2025). Yoshiki Kuramoto used the theory of coupled oscillators to show turbulence in fluid dynamics (Kuramoto, 1984), suggestive of how turbulence could be important not only for energy transfer but for efficient information transfer. Specifically, this framework defines the Kuramoto local order parameter, representing a spatial average of the complex phase factor of the local oscillators weighted by the coupling. Thus, the Kuramoto local order parameter gives the level of synchronization of the local phases around a specific point. This is what we call *Kuramoto vorticity*. Importantly, the level of amplitude turbulence is defined as the standard deviation of the modulus of Kuramoto vorticity and can be applied to the empirical data of any physical system. Remarkably, brains also were also found to exhibit a similar turbulent power law, strongly suggesting the presence of a cascade of efficient information processing across scales (Deco & Kringelbach, 2020; Deco, Leibana Garcia et al., 2023; Deco et al., 2025).

As can be appreciated from Figure 2, both spirals and Kuramoto vorticity show the same phenomenon, namely the local rotation of the moving flow, albeit in different complementary ways. Specifically, the Kuramoto vorticity in the 2D vertex space, *R*(***x***, *t*), is defined by:

$$R\left(x,t\right)e^{i\theta \left(x,t\right)}=\int_{\mathbb{N}\left(x\right)}e^{i\varphi \left(y,t\right)}dy$$ (4)

where *φ*(***y***, *t*) is the local phase of the BOLD signal in the 2D vertex space at point ***y***. The integral range ℕ(***x***) is defined by a circle of radius of 4 mm around ***x***. We measure amplitude turbulence by first defining the Kuramoto local order parameter and then taking the standard deviation of the modulus across time and space (Kawamura et al., 2007). Thus, the Kuramoto turbulence, *T*_*Kuramoto*_, is defined as the standard deviation of the Kuramoto vorticity across space and time:

$$T_{\text{Kuramoto}}=\langle R{\left(n,t\right)}^{2}\rangle -{\langle R\left(n,t\right)\rangle }^{2}$$ (5)

where the brackets 〈 〉 denotes average across vertex space and time.

In order to directly compare Spiral vorticity and Kuramoto vorticity, we move from vertex space to the more coarse-grained Schaefer1000 parcellation by averaging the values of the vertices within each parcel in the subset of 100 HCP participants used for the spiral vorticity.

### Kuramoto Vorticity Whole-Brain Model

A key finding of the coarse-grained partition methodology is the derivation of a model reduction of a coupled system of Kuramoto oscillators (Snyder et al., 2020). One of the most well-known examples of this kind of model reduction is the groundbreaking work by Ott and Antonsen (Ott & Antonsen, 2008), who demonstrated that in the limit of an infinite number of oscillators, the overall synchronization level follows an autonomous ODE. In neuroscience, research has robustly demonstrated that a system of Kuramoto oscillators coupled through the structural anatomical connectivity matrix is able to achieve an excellent fitting of functional brain activity obtained with MEG (Cabral et al., 2014).

Given that the vortices are fundamental elements of the brain dynamics associated with the processing and computation of cognition, it would be convenient to derive a direct reduced model of the vortical activity emerging from an underlying Kuramoto whole-brain model. Furthermore, if the underlying base Kuramoto whole-brain model is defined in a fine parcellation (in our case, the Schaefer1000), the reduced model of the vortical activity will conveniently be defined in a more coarse parcellation (here Schaefer100).

Let us consider a Kuramoto whole-brain model in the Schaefer1000 parcellation. The models consist of a network of *M* = 1,000 coupled phase oscillators, where the connections are defined by the anatomical connectivity matrix, which was estimated using dMRI data. This model assumes that oscillators interact based on their phase differences. Let $\hat{\varphi }\left(i,t\right)$ represent the phase of the *i-th* oscillator at time *t*. We will use the circumflex hat to denote all the variables related to the Schaefer1000 parcellation with respect to the variables in vertex space (with circumflex). The phases evolve according to the following set of coupled differential equations:

$$\frac{d\hat{\varphi }\left(i,t\right)}{dt}=\omega_{i}+G\sum_{j=1}^{M}C_{ij}\sin\left(\hat{\varphi }\left(j,t\right)-\hat{\varphi }\left(i,t\right)\right)$$ (6)

where *ω_i_* is the natural frequency of the *i-th* oscillator, while *C*_*ij*_ is the anatomical structural connectivity matrix obtained by dMRI in the Schaefer1000 parcellation (see above), and *G* represents the global coupling strength. The natural frequency of oscillations for each ROI was estimated from the peak of the power spectra estimated from their BOLD in the frequency band 0.008–0.08 Hz. The interaction between two oscillators, *i* and *j*, is governed by the sine of their phase difference. This interaction promotes synchronization, since an oscillator lagging behind (that is $\hat{\varphi }\left(j,t\right)-\hat{\varphi }\left(i,t\right)>0$) speeds up, while one ahead (that is $\hat{\varphi }\left(j,t\right)-\hat{\varphi }\left(i,t\right)<0$) slows down. This model was numerically integrated using Euler’s method with a time step of 0.01 (equivalent to 10 ms).

One interesting order parameter of the system is the global Kuramoto order parameter, ${\hat{R}}_{G}$, given by:

$$z\left(t\right)={\hat{\;R}}_{G}\left(t\right)e^{i{\hat{\theta }}_{G}\left(t\right)}=\frac{1}{M}\sum_{p=1}^{M}e^{i\hat{\varphi }\left(p,t\right)}$$ (7)

where ${\hat{R}}_{G}\in \left[0,1\right]$ is the global synchrony and ${\hat{\theta }}_{G}\in \left[0,2\pi \right]$ is the average phase. If all phases are equal then ${\hat{R}}_{G}=1$, and if the phases are spread uniformly over the unit circle, then ${\hat{R}}_{G}\approx 0$. Thus ${\hat{R}}_{G}$ is a natural measure of synchronization. It is interesting to note that in the case of *C*_*ij*_ = 1/*M*, the global Kuramoto order parameter follows a closed equation. Assuming that *ω_i_* is Cauchy distributed, i.e., *ω_i_*~*f*(*ω*) where *f* is the Cauchy probability density function with mode Ω and width *δ*, and that the coupling is mean field, then ${\hat{R}}_{G}\left(t\right)$ evolves according to a Stuart–Landau equation,

$$\frac{dz\left(t\right)}{dt}=\left(i\Omega -\delta +\frac{G}{M}\right)z\left(t\right)-\frac{G}{2}z\left(t\right){\left|z\left(t\right)\right|}^{2}$$ (8)

The last equation shows that *z* = 0 is always a solution but undergoes a pitchfork bifurcation at *G*_*critical*_ = 2*δ*, when a new solution with RˆG=1−2δG, and $\frac{d{\hat{\theta }}_{G}\left(t\right)}{dt}=\Omega$ appears, representing partial synchrony that becomes global synchrony (i.e., ${\hat{R}}_{G}=1$) as *G* → ∞.

### Partitioning: Whole-Brain Model in Vortex Space

Importantly, a very similar analytical derivation can be carried out for the case where oscillators are not all coupled to each other but are instead grouped into subsets, with the coupling strength between any two oscillators depending on the subsets they belong to. In other words, a close equation can be found for the local Kuramoto order parameters evolution, that is for the dynamics of the vortex space, defined in a partition of the original system. Here, the original system of Kuramoto oscillators was defined above in the Schaefer parcellation *M* = 1,000.

We define a partition of that parcellation by adopting for the local Kuramoto order parameters a Schaefer parcellation *N* = 100. Let ℘ = (ℙ(1), …., ℙ(*N*)) represent a partition of the index set [1, …, *M*] and let *K* be a *N* × *N* matrix of coupling strengths. Following Snyder and colleagues (Snyder et al., 2020), such a modular system can be expressed as:

$$\frac{d\hat{\varphi }\left(i,t\right)}{dt}=\omega_{i}+\sum_{\sigma \prime =1}^{N}\frac{K_{\sigma \sigma \prime }}{|P\left(\sigma^{\prime }\right)|}\sum_{j\subset \mathbb{P}\left(\sigma^{\prime }\right)}\sin\left(\hat{\varphi }\left(j,t\right)-\hat{\varphi }\left(i,t\right)\right)$$ (9)

The same mathematical framework can be used to show that if, for each *σ*, the natural frequencies *ω_i_* ∣ *i* ⊂ ℙ(*σ*) are distributed according to a Cauchy distribution with mode Ω*_σ_* and width *δ_σ_*, then the cluster order parameters *z_σ_*(*t*), defined by

$$z_{\sigma }\left(t\right)=r_{\sigma }\left(t\right)\;e^{i\vartheta_{\sigma }\left(t\right)}=\frac{1}{|P\left(\sigma \right)|}\sum_{p\subset \mathbb{P}\left(\sigma \right)}e^{i\hat{\varphi }\left(p,t\right)}$$ (10)

satisfy a coupled Stuart-Landau equation of the form:

$$\frac{dz_{\sigma }\left(t\right)}{dt}=\left(i\Omega_{\sigma }-\delta_{\sigma }\right)z_{\sigma }\left(t\right)+\frac{1}{2}\sum_{\sigma^{\prime }=1}^{N}K_{\sigma \sigma^{\prime }}\left(z_{\sigma^{\prime }}\left(t\right)-z_{\sigma^{\prime }}^{*}\left(t\right)z_{\sigma^{\prime }}^{2}\left(t\right)\right)$$ (11)

In this context, the natural emergence of last equation suggests that in the Kuramoto model, groups of oscillators can collectively behave as a single oscillator. This implies that there exists a renormalization process for coupled oscillator systems that keeps them within the same model framework. Note, that the cluster order parameters *z_σ_*(*t*) are the Kuramoto vorticity on the Schaefer1000 parcellation defined by averaging spatially the complex phase factor of the local phases $\hat{\varphi }\left(p,t\right)$ from the BOLD time series signals defined in this parcellation. The Kuramoto vorticity, *z_σ_*(*t*) reflect the level of synchronization of the local phases in a given partition *σ* (in the Schaefer100 parcellation). Equation 11 is thus an explicit model of the vortex space.

Finally, in order to fit the vortex model to the empirical vortex data, we use for the simulations of the vortex space in the Schaefer100 parcellation following equation:

$$\frac{dz_{\sigma }\left(t\right)}{dt}=\left(<i\omega_{i}{>}_{i\subset \mathbb{P}\left(\sigma \right)}-\delta_{\sigma }\right)z_{\sigma }\left(t\right)+G_{\text{vortex}}\sum_{\sigma^{\prime }=1}^{N}K_{\sigma \sigma^{\prime }}\left(z_{\sigma^{\prime }}\left(t\right)-z_{\sigma^{\prime }}^{*}\left(t\right)z_{\sigma }^{2}\left(t\right)\right)$$ (12)

where *G*_*vortex*_ is a free parameter expressing the global coupling in vortex space, and *K*_*σσ*′_ is the anatomical structural connectivity matrix obtained by dMRI in the Schaefer100 parcellation (see above). In the simulations, we used *δ_σ_* = 0.001 and added Gaussian noise with and standard deviation of 0.01. The diagonal elements of *K_σσ_* were set to $\sum_{\sigma^{\prime }=1,\sigma \neq \sigma^{\prime }}^{N}K_{\sigma \sigma^{\prime }}$ in order to recover the usual form of the Hopf whole-brain model (Deco et al., 2017), which provides a good level of fitting as shown in the results. Consequently, the parameter selection was empirical or according to the optimal results demonstrated in previous works using the Hopf whole-brain model (Deco & Kringelbach, 2025; Deco et al., 2017).

When we optimize the Kuramoto model, we fit the free parameter *G* (global coupling in the Schaefer1000 parcelation and BOLD space) by fitting the PLV (Phase Locking Value of the signal in the Schafer 1000 parcellation, using our previously published methods (Ponce-Alvarez et al., 2015), that is, ${PLV}_{ij}=|\frac{1}{T}\sum_{t=1}^{T}e^{i\left(\hat{\varphi }\left(i,t\right)-\hat{\varphi }\left(j,t\right)\right)}|$ being *T* the maximal measured or simulated time, 1200 TR) and after finding the optimal *G*, we compute the associated vortex space with Equation 10 to compute the FC in vortex space as indicated below (Equation 14).

In order to directly compare the accuracy of the Kuramoto phase model with the Hopf vortex model, we compare the simulation FC in vortex space with the empirical FC vortex space.

### Capturing Vortex Interactions

In order to explicitly quantify the Kuramoto vortical interactions in the Schaefer100 parcellation, we extend Equation 12 to optimize instead the global conductivity parameter *G*_*vortex*_, the single paired conductivity parameters. The key idea is to fit the empirical FC of the Kuramoto vortices by the model described in Equation 12. This provides the GEC of the vortex space (GEC Vortex), which is the effective weighting of the existing anatomical connectivity coupling the vortices in the Schaefer100 parcellation. Note that this is an extension of the classic concept of effective connectivity (Friston et al., 2003), where (a) GEC is generative, using the whole-brain model to adapt the strength of existing anatomical connectivity (i.e., the effective conductive values of each fiber) and (b) the optimization target for GEC is in vortex space, that is the FC of the Kuramoto vortices. In other words, creating a whole-brain model in the vortex space of the empirical neuroimaging data provides direct access to determining the interactions between vortices.

We optimized GEC Vortex between brain regions by comparing the output of the vortex model:

$$\frac{dz_{\sigma }\left(t\right)}{dt}=\left(<i\omega_{i}{>}_{i\subset \mathbb{P}\left(\sigma \right)}-\delta_{\sigma }\right)z_{\sigma }\left(t\right)+\sum_{\sigma^{\prime }=1}^{N}E_{\sigma \sigma^{\prime }}\left(z_{\sigma^{\prime }}\left(t\right)-z_{\sigma^{\prime }}^{*}\left(t\right)z_{\sigma }^{2}\left(t\right)\right)$$ (13)

with the empirical measures of FC in vortex space. Let us denote by ${FV}_{\sigma \sigma^{\prime }}^{\text{model}}$ and ${FV}_{\sigma \sigma^{\prime }}^{\text{empirical}}$ the simulated and empirical FC in vortex space, respectively. In our definition of FC in vortex space, we considered the conjugated correlations as following:

$${FV}_{\sigma \sigma^{\prime }}=\frac{<z_{\sigma }-<z_{\sigma }{>}_{t}{>}_{t}<z_{\sigma \prime }-<z_{\sigma \prime }{>}_{t}{>}_{t}\;}{\sqrt{<{\left(z_{\sigma }-<z_{\sigma }{>}_{t}\right)}^{2}{>}_{t}}\;\sqrt{<{\left(z_{\sigma \prime }-<z_{\sigma \prime }{>}_{t}\right)}^{2}{>}_{t}}}\cos\left(\vartheta_{\sigma }-\vartheta_{\sigma \prime }\right)$$ (14)

where the brackets 〈 〉*_t_* denotes average across time. In the model case, the Kuramoto vortices are simulated by using Equation 13. In the empirical case, the Kuramoto vortices are computed by using the Hilbert transform of the filtered empirical BOLD data (second-order zero-phase Butterworth filter: 0.008 Hz < f < 0.08 Hz) in the Schaefer1000 parcellation and defined as in Equation 10, but instead using the empirical extracted phases.

Using a heuristic gradient algorithm, we proceed to update the GEC such that the fit is optimized. In order to work only positive values for the algorithm, all values are transformed into a mutual information measure (assuming Gaussianity). More specifically, the updating uses the following form:

$$E_{\sigma \sigma^{\prime }}=E_{\sigma \sigma^{\prime }}+\epsilon \left({FV}_{\sigma \sigma^{\prime }}^{\text{empirical}}-{FV}_{\sigma \sigma^{\prime }}^{\text{model}}\right)$$ (15)

The model was run repeatedly with the updated GEC until the fit converges toward a stable value. In each iteration step, as before, the diagonal elements of *E_σσ_* were set to $\sum_{\sigma^{\prime }=1,\sigma \neq \sigma^{\prime }}^{N}E_{\sigma \sigma^{\prime }}$.

We initialized using the anatomical connectivity (obtained with probabilistic tractography from dMRI) and only update known existing connections from this matrix (in either hemisphere). However, there is one exception to this rule, which is that the algorithm also updates homologue connections between the same regions in either hemisphere, given that tractography is known to be less accurate when accounting for this connectivity. We used *ε* = 0.01, and continue until the algorithm converges. For each iteration, we compute the model results as the average over as many simulations as there are participants. In the simulations, we used *δ_σ_* = 0.001 and added Gaussian noise with a standard deviation of 0.01.

### Support Vector Machine Used For Condition Classification

We used an SVM with polynomial kernels as implemented in the MATLAB function *fitcecoc*. The function returns a fully trained, multiclass, error-correcting output codes model. This is achieved using the predictors in the input with class labels. The SVM used inputs of the 100 × 100 matrices of model GEC Vortex, while the output was two classes corresponding to the conditions. We used the output from all 971 HCP participants used for generalization, subdivided into 90% training and 10% validation, repeated and shuffled 1,000 times. To evaluate whether the observed accuracy values were statistically meaningful, we generated 1,000 surrogate SVM classifiers that used the same feature set (i.e., GEC Vortex matrices) but with randomly shuffled class labels. An empirical *p* value was then computed by counting how often the accuracy of these label-shuffled classifiers exceeded that of the true classifier.

### Computing *g*-Factor Measure of Intelligence

The *g*-factor was computed using a MATLAB adaptation of the procedure described by Dubois and colleagues (Dubois et al., 2018) to perform factor analysis of the scores on ten relevant cognitive scores from the behavioral psychometric battery used to assess each HCP individuals. This procedure derives the *g*-factor measure of intelligence, which is a standard used in the field of intelligence research.

### CPM

For using brain patterns to predict behavior, we implemented CPM using the standard procedure of previous studies (Greene et al., 2018; Shen et al., 2017). In particular, we compared the GEC Vortex and FC BOLD matrices. The former characterizes the interactions between turbulent vortices, while the latter characterizes the correlation of BOLD signals. We carried this out to predict the composite *g*-factor and four of the most important underlying task scores from different cognitive domains. First, we performed a feature selection on the full data set for the dimensionality of the GEC Vortex/FC feature space. Each GEC Vortex/FC edge was correlated with the behavioral measure using Pearson correlation. Edges exhibiting an absolute correlation magnitude greater than a predefined threshold (|*r*| > 0.17) were selected as candidate predictive features. We then standardized the selected features were (z-scored) across subjects prior to model training. In accordance with CPM conventions, standardization was performed once on the full dataset rather than within each training fold. The same standardized predictors were used for all subsequent cross-validation procedures.

Model performance was evaluated using 100 repetitions of a 10-fold outer cross-validation framework. In each repetition, the data were randomly partitioned into 10 folds. For a given outer fold, training data were used to determine the optimal regularization parameter for elastic net regression. Specifically, a second, inner 10-fold cross-validation was conducted within the training set only, using the elastic net penalty parameter *α* = 0.01. The value of the L1/L2 regularization parameter (*λ*) minimizing mean square error in the inner loop was selected.

After identifying the optimal *λ*, a final elastic net model was refitted on the entire training set using this *λ*. This model was then used to generate out-of-sample predictions for the held-out test subjects in the outer fold. This procedure was repeated across all 10 folds, ensuring that every subject received a prediction generated from a model trained on data not containing that subject. The entire nested cross-validation procedure was repeated 100 times to assess variability in model performance. Finally, for each repetition, predictive performance was quantified using two complementary metrics computed across all subjects: (a) Pearson correlation between observed and predicted behavioral scores; (b) cross-validated coefficient of determination (*R*^2^), computed as:

$$R^{2}=1-\frac{\sum_{i=1}^{N}{\left(y_{i}-\hat{y_{i}}\right)}^{2}}{\sum_{i=1}^{N}{\left(y_{i}-\bar{y}\right)}^{2}}$$ (16)

## ACKNOWLEDGMENTS

G.D. is supported by Grant PID2022-136216NB-I00 funded by MICIU/AEI/10.13039/ 501100011033 and by “ERDF A way of making Europe,” ERDF (European Regional Development Fund European Union), EU, Project NEurological MEchanismS of Injury, and Sleep-like cellular dynamics (NEMESIS) (ref. 101071900) funded by the EU ERC Synergy Horizon Europe, and AGAUR research support grant (ref. 2021 SGR 00917) funded by the Department of Research and Universities of the Generalitat of Catalunya. Y.S.P. is supported by was supported by the project NEMESIS (ref. 101071900) funded by the EU ERC Synergy Horizon Europe. M.L.K. is supported by the Centre for Eudaimonia and Human Flourishing (funded by the Pettit and Carlsberg Foundations) and Center for Music in the Brain (funded by the Danish National Research Foundation, DNRF117). The funders had no role in study design, data collection and analysis, decision to publish, or preparation of the manuscript.

## AUTHOR CONTRIBUTIONS

Gustavo Deco: Conceptualization; Data curation; Formal analysis; Funding acquisition; Investigation; Methodology; Project administration; Resources; Software; Supervision; Validation; Visualization; Writing – original draft; Writing – review & editing. Yonatan Sanz Perl: Conceptualization; Data curation; Formal analysis; Funding acquisition; Investigation; Methodology; Project administration; Resources; Software; Supervision; Validation; Visualization; Writing – original draft; Writing – review & editing. Jianfeng Feng: Writing – review & editing. Morten L. Kringelbach: Conceptualization; Data curation; Formal analysis; Funding acquisition; Investigation; Methodology; Project administration; Resources; Software; Supervision; Validation; Visualization; Writing – original draft; Writing – review & editing.

## FUNDING INFORMATION

Grant PID2024-162576NA-I00 funded by MICIU/AEI/10.13039/501100011033 and by “ERDF A way of making Europe”.

## DATA AND CODE AVAILABILITY

The open-source MATLAB code can be found here: https://github.com/decolab/TTB_modellingvortexinteraction.

## TECHNICAL TERMS

Turbulence: Highly variable and dynamic patterns of system exhibiting local synchronizations, characterized by significant fluctuations across space and time.
Whole-brain model: A causal model of brain activity constructed using the local dynamics of each region in a parcellation coupled through the underlying anatomical connectivity and fitted to the empirical whole-brain data.
GEC: A connectivity matrix of a given parcellation using whole-brain modeling to fit the underlying functional connectivity of the brain data using an optimized version of the topology of the structural connectivity.
Global Kuramoto order parameter: A measure of global synchronization based on the phases of the elements in a system.
Local Kuramoto order parameter: A measure of local synchronization.
Oscillator: Dynamical systems exhibiting periodic behavior over time, characterized by the regular evolution of its state variables (such as phase and amplitude) around an equilibrium point.
Navier–Stokes equation: An equation governing the local dynamics of a fluid.
Spiral: A measure of vorticity quantified by the curl of the flow.
Vorticity: Local rotation of motion of a fluid around a specific point. Here, the level of local synchronization around a specific spatial region.
GEC vortex: The GEC in vortex space characterizing the interactions between turbulent vortices.
